# Transforming health systems: Mapping the landscape with 172 regional cross-sector partnerships in the Netherlands

**DOI:** 10.1016/j.hpopen.2026.100192

**Published:** 2026-10-23

**Authors:** Sam Hundersmarck, Miel Vugts, Annefrans van Ede, Marc Bruijnzeels, Natascha van Vooren

**Affiliations:** a Department of Population Health and Health Services Research, National Institute for Public Health and the Environment (RIVM), Postal address: RIVM, P.O. Box 1, Bilthoven, 3720 BA, the Netherlands; b Health Campus The Hague/ Department of Public Health and Primary Care, Leiden University Medical Center, Spui University Campus, Spui 5, The Hague, 2511 BL, the Netherlands

**Keywords:** Cross-sector partnerships, Regional collaboration, Integrated care, Population health, Health system transformation, Network governance, Netherlands

## Abstract

**Background:**

Regional collaborative approaches have become an important feature of health system transformation across the globe, including the Netherlands. While cross-sector partnerships (CSPs) are increasingly relied upon to coordinate organizations around population health and wellbeing goals, empirical insight into how such partnerships are configured at scale within specific policy contexts remains limited.

**Objective:**

This study aims to map the national landscape of CSPs in the Netherlands by identifying distinct partnership configurations and examining how these relate to determinants for sustainable health and wellbeing system development.

**Methods:**

A cross-sectional study was conducted using data from two rounds (2023–2024) of a national survey of CSPs (*n* = 172). Structural and strategic characteristics were analyzed descriptively and used in a latent class analysis to identify distinct CSP configurations, which were subsequently compared across determinants for sustainable health and wellbeing system development.

**Results:**

Two configurations were identified: (1) broadly oriented CSPs, characterized by larger scale, wider population scope, and broader prevention focus; and (2) smaller, targeted CSPs focusing on specific populations or care pathways. Differences mainly reflect variation in strategic orientation and associated coordination demands, with broadly oriented CSPs more often coordinating multiple networks and organizations and aligning with current policy agendas. Despite these differences, both configurations showed similar patterns across the determinants, with trust, leadership, and shared commitment relatively well established, but accountability structures, financial arrangements, and data availability less developed.

**Conclusions:**

The findings indicate that Dutch CSPs take distinct, strategically varied configurations, but nonetheless face broadly similar challenges in moving toward more structurally embedded collaboration within their shared policy context.

**Research in con text:**

**What is already known about the topic?**

Cross-sector partnerships (CSPs) have emerged as a central feature of health system transformation, but their organization across health system contexts remains poorly understood. Evidence on outcomes is mixed, while studies consistently identify relational and structural conditions that support collaboration. Larger-scale studies, primarily from the United States, show that CSPs are diverse and face recurring challenges related to financing, coordination, and outcome measurement.

**What does this study add to the literature?**

This study provides the first empirical, national-level overview of CSPs in the Netherlands, identifying distinct but coexisting configurations within a shared policy context. While CSPs differ in strategic orientation and coordination demands, they display similar patterns across determinants for sustainable health and wellbeing system development.

**What are the policy implications?**

Policymakers should recognize that CSPs operate through multiple configurations with differing coordination demands. At the same time, shared developmental challenges, particularly in accountability formalization, financial arrangements, and data availability highlight areas where support at the partnership level may need to be complemented by support at the system level to enable further consolidation and long-term sustainability.

## Highlights

• Dutch cross-sector partnerships (CSPs) show substantial organizational variation.
• Broadly oriented and more targeted partnerships coexist within the same context.
• Both show similar patterns across principles for sustainable health and wellbeing systems development.
• Trust, leadership, and commitment are relatively well established.
• Accountability, payment structures, and data availability remain more underdeveloped.

## 1 Background

As in other countries worldwide, the Netherlands has seen growing attention to regional collaborative approaches in the organization of health and wellbeing services in response to shared challenges, including population ageing, workforce shortages, widening health inequalities, and rising healthcare expenditures 1., 2., 3., 4.. However, while regional collaboration is becoming a cornerstone of Dutch health policy, surprisingly little is known about how it is organized across the country. The growing reliance on regional partnerships as a coordination mechanism appears to have outpaced empirical understanding of how they are configured across the country or how they differ from one region to another.

Many challenges to population health, such as health inequalities, extend across areas of responsibility and require the involvement of multiple stakeholders with different interests and institutional logics 5., 6., 7., 8.. Often described as wicked problems, these challenges are characterized by interdependence, competing values, and ambiguous boundaries, creating a need for collective action across organizations and sectors 7., 8.. Such efforts can, however, be complicated by how roles and responsibilities are distributed and organized across the system 9., 10.. Internationally, countries have developed different approaches to organizing regional collaboration, including Integrated Care Systems (ICSs) in England, Ontario Health Teams (OHTs) in Canada, regionally organized integrated care initiatives in Belgium, wellbeing services counties (WCSs) in Finland, and Regional Partnership Boards (RPBs) in Wales 11., 12., 13., 14., 15.. These approaches differ in their organization and scope, with some primarily focusing on coordinating and integrating healthcare delivery, and others involving a broader range of sectors and organizations addressing population health and wellbeing. Despite these differences, they share an emphasis on coordinating organizations around the needs of defined populations and improving population health.

In the Netherlands, regional collaboration takes place within a highly decentralized health system characterized by managed competition, in which regulation, payment, and service delivery are distributed across autonomous actors and organized through separate policy sectors, including healthcare, social support, long-term care, and public health, with distinct roles and payers 16., 17.. Moving toward a broader health and wellbeing system therefore entails active coordination across these traditionally separated sectors. Within this context, local actors have increasingly adopted cross-sector partnerships (CSPs): purpose-oriented networks of three or more autonomous organizations working toward shared health and wellbeing goals 18., 19..

While CSPs provide a structure for bringing organizations together around shared population health challenges, their formation does not in itself resolve the underlying coordination challenges 5., 6., 20., 21., 22., 23., 24.. Collaborative arrangements can themselves introduce additional demands, as involving multiple autonomous organizations can delay decision-making, create ambiguity in roles and responsibilities, and increase the number of relationships that need to be managed 9., 25.. Studies have consistently highlighted that effective collaboration among diverse organizations depends on relational factors, such as trust and leadership, and structural conditions, including appropriate governance arrangements and adequate resources 26., 27., 28., 29., 30.. In related work, Van Vooren et al. [31] identified eight guiding principles for sustainable health and wellbeing system development, providing a framework for the conditions considered important for establishing and sustaining collaboration across organizations and sectors. These principles comprise: (1) shared commitment, referring to a common vision and collective ambition; (2) mutual understanding and trust, including understanding differences in roles, values, and working practices; (3) accountability and governance, concerning clear roles, responsibilities, and decision-making; (4) alignment with policy, including political support and engagement with policy actors; (5) financial arrangements, including payment models and incentives that support shared goals; (6) data and learning, concerning the infrastructure and learning processes needed for monitoring and improvement; (7) community input and involvement, referring to the involvement of citizens and communities in priorities and decision-making; and (8) stakeholder representation and leadership, concerning the involvement of appropriate stakeholders and leadership to guide collaboration.

Despite growing understanding of the conditions that support cross-sector collaboration, evidence that such collaboration translates into improved health and service outcomes remains limited and mixed [6]. Moreover, there is less systematic understanding of how the organizational characteristics of CSPs combine into different forms of collaboration, and whether these forms entail different coordination challenges and depend on different conditions. This gap is reinforced by limitations of the existing empirical literature, which consists primarily of case-based analyses or small-sample studies that provide valuable insights into how collaboration unfolds in practice and the challenges partnerships encounter, but offer only partial insight into how partnerships vary and function across different settings 6., 20., 26., 32.. As pointed out by Alderwick et al. [6], existing research encompasses widely differing forms of collaboration, which are often studied together without consistent descriptions of their organizational characteristics. This makes it difficult to systematically analyze and compare these partnerships across contexts and to identify recurring patterns in how CSPs are organized and strategically oriented. A clearer understanding of these patterns can provide a basis for examining their development, the challenges they encounter, and how sustainable cross-sector collaboration can be supported.

A limited number of studies, primarily in the United States, have examined cross-sector collaborations at a larger scale, providing broader insight into their functioning. These show that, although the initiatives are widespread and diverse, they face common challenges related to financing, coordination, and outcome measurement33., 34., 35.. Nonetheless, as the opportunities and constraints for CSP organization and development are shaped by the health system and policy context in which they operate, findings from predominantly one context may provide only a partial understanding of their characteristics and developmental challenges6., 21., 36.. Comparable analyses in other settings are therefore important for expanding the empirical basis and providing insight into whether CSPs with similar configurations face comparable challenges across contexts, thereby strengthening the basis for cross-context learning. Such insights may also help identify gaps and priorities for further research and policy attention.

The Netherlands offers a complementary setting in which the growing emphasis on cross-sector collaboration around population health has resulted in a rich landscape of CSPs, including both established collaborations and more recent, policy-driven partnerships. The latter are supported by national multistakeholder agreements, most notably the *Integraal Zorgakkoord* (IZA)[37], introduced in 2022, alongside the *Gezond en Actief Leven Akkoord* (GALA)[38], introduced in 2023. These agreements were developed by representatives of national and local government, health insurers, healthcare providers, and other stakeholders, including organizations from public health and social care. They aim to strengthen regional and cross-sector collaboration to support a shift from care toward prevention, health, and wellbeing. Their implementation is supported and incentivized through several policy instruments, including regional plans that establish shared priorities and dedicated funding arrangements. Under the IZA, regional stakeholders can apply for transformation funding through plans assessed by the two market-leading health insurers in their region, while the national government makes funding available to municipalities through a specific-purpose grant to support local health promotion and prevention initiatives under GALA. Progress is monitored nationally by organizations such as the National Institute for Public Health and the Environment (RIVM) [39].

Together, the established collaborations and newer partnerships supported by the 10.13039/501100011669IZA and GALA contribute to a diverse and evolving landscape of CSPs, raising questions about their current configurations and how they are positioned in relation to conditions considered important for sustainable collaboration.

Accordingly, this study addresses three questions:

1. How are CSPs in the Netherlands configured in terms of their structural and strategic characteristics?
2. What patterns can be identified across these configurations?
3. How do these CSP configurations relate to the extent to which guiding principles for sustainable health and wellbeing system development are reflected in practice?

## 2 Methods

### 2.1 Study design

This study used a cross-sectional design to examine CSPs in the Netherlands. Survey data were collected between September and December 2023 (round one) and between November 2024 and January 2025 (round two). The data were analyzed descriptively to map structural and strategic characteristics of the CSPs. Subsequently, a latent class analysis (LCA) was conducted to identify distinct CSP configurations, which were then compared across determinants relevant to sustainable health and wellbeing system development.

### 2.2 Data source

This study draws on the data of the first two rounds (2023–2024) of a national, annually recurring survey targeting a broad convenience sample of CSPs in the Netherlands. The survey was conducted and developed by the RIVM as part of a multi-year evaluation initiative commissioned by the Ministry of Health, Welfare and Sport (VWS) and designed to build on earlier national monitoring and evaluation efforts 19., 40., 41.. Its design drew on validated instruments and literature on factors associated with the effectiveness and functioning of purpose-oriented networks, as well as guiding principles for sustainable health and wellbeing system development 20., 31., 42., 43.. For reference, the full questionnaire is provided in Appendix A.

### 2.3 Study sample

The survey was distributed digitally to program managers and coordinators of active CSPs across the Netherlands through a combination of direct email invitations and open dissemination channels, including newsletters, LinkedIn posts, and relevant learning and policy networks. In both survey rounds, two reminder emails were sent to non-responding contacts.

Potential partnerships were identified through multiple sources, including public registries, existing RIVM contact lists, professional networks, and previous inventories of regional collaboration initiatives. In 2023, approximately 246 initiatives were contacted directly by email, supplemented by invitations to initiatives participating in relevant national funding programs (approximately 176 additional projects). In 2024, an updated database of approximately 533 potential initiatives was used.

To distinguish CSPs, as ongoing, purpose-oriented collaborative arrangements, from individual projects or interventions, informal collaborations, or interorganizational networks without a clear population focus, CSPs included in this study were required to meet the following inclusion criteria:

• The partnership consists of three or more organizations that share a joint ambition to improve care or support for residents within a specific geographic area in the Netherlands.
• Participating organizations operate across at least two distinct policy sectors:
  o Social care (as covered by the Social Support Act, Youth Act, Public Health Act, or Participation Act)
  o Long-term care (Long-term Care Act)
  o Curative healthcare (Health Insurance Act)
• The initiative targets a defined territory, ranging from multiple neighborhoods to one or more adjacent municipalities or regions, focusing on improving population health.
• The partnership is intended to last for more than one year and is actively engaged in at least two concrete collaborative activities (e.g., projects, interventions) aimed at addressing the needs and functioning of residents or patients.

The final criterion reflects the study's focus on partnerships in which collaboration extends beyond the delivery of a single project or intervention and is oriented toward a longer-term shared purpose. Focusing on a one-off joint activity is less likely to provide insight into how organizations organize and sustain coordination around shared goals within an ongoing partnership. This also helps ensure that the partnerships themselves, rather than individual collaborative activities, constituted the unit of analysis.

## 3 Data collection

For answering the first two research questions, variables were selected a priori based on their conceptual relevance to distinguishing partnership configurations. As this study is based on a secondary analysis of survey data, the selection of variables was necessarily constrained by the items available in the original questionnaire. The selected variables capture complementary aspects of CSPs' structural and strategic characteristics, including the scale and composition of the partnership, the population addressed, and the breadth and orientation of its goals. Drawing on Provan and Kenis [9], the number of participating organizations is relevant to the coordination demands associated with managing relationships and activities, organizational composition to the different responsibilities and perspectives that need to be aligned, and the population addressed and goal orientation to the scope and nature of the shared task around which coordination is organized. Collectively, these characterize what CSPs seek to accomplish, for whom, with whom, and at what scale.

Examining these characteristics individually provides insight into variation across CSPs, but not into whether they combine in recurring patterns. LCA was therefore used to identify distinct configurations based on patterns across these characteristics. To support model stability and the identification of meaningful and interpretable classes, the analysis focused on a limited set of conceptually distinct variables, which were constructed from one or more survey items and, where necessary, recoded or aggregated to create distinctive categorical indicators (Table 1). The resulting configurations provide a basis for systematically characterizing and comparing different forms of CSPs.

**Table 1:** Indicators and their operationalization for LCA.

**Indicato r**	**LCA operationalization**
Number of participating organizations	Categorized into 1–10 (1), 11–25 (2), and more than 25 (3)
Target population size	Categorized into <10,000 (1), 10,000–100,000 (2), and ≥ 100,000 individuals (3)
Target population: Residents (regardless of care or support use)	Unchanged; yes/no
Prevention orientation	Binary indicator coded as “yes” (2) if the CSP reported a focus on either universal prevention, selective prevention, or both; otherwise coded as “no” (1)
Outcome domains	Summed across Quintuple Aim items; recoded into fewer than 4 domains (1) versus 4–5 domains (2)
Municipal public health service involvement	Unchanged; yes/no

Regarding the third research question, the determinants and underlying indicators were selected for their conceptual relevance to the guiding principles for sustainable health and wellbeing system development formulated by Van Vooren et al [31]. Accordingly, these were organized into eight distinct determinant categories: governance and accountability, trust, leadership, data and learning, financial arrangements, target population engagement, alignment with policy, and shared commitment. The determinants were operationalized using one or more survey items, as detailed in Table B1 (Appendix B), with most indicators derived directly from individual items, except for trust and leadership (composite measures) and alignment with GALA policy (aggregated indicators).

In addition, perceived sufficiency of non-financial resources was included as an indicator of partnerships' capacity to support and sustain collaborative processes, based on broader literature on collaboration and purpose-oriented networks identifying resource sufficiency as an important factor associated with partnership effectiveness 9., 20., 42., 43.. This determinant was operationalized using instruments developed by Cramm et al. [42], assessing perceived sufficiency across multiple resource domains, including skills and expertise, data and information, target population relationships, and convening power. Although these domains partly overlap with several guiding principles, this determinant captures whether partnerships perceive these resources as sufficiently available to support their collaboration in practice.

## 4 Data analysis

### 4.1 Structural and strategic characteristics and latent-class analysis

Structural and strategic characteristics of CSPs were summarized using descriptive statistics, including frequencies, percentages, and medians with interquartile ranges (IQR), where appropriate. These analyses were conducted for the full sample to provide an overview of variation in partnership scale, population reach, and strategic orientation.

To identify potentially distinct subgroups of CSPs based on shared structural and strategic characteristics, a Latent Class Analysis (LCA) was conducted [44]. Prior to the analysis, missing data were handled using multiple imputation (five datasets) with the *mice* package in R, applying ordinal regression for ordinal variables and multinomial regression for nominal variables [45]. Convergence was assessed using trace plots. The LCA was then conducted for each imputed dataset using the *poLCA* package [46] in R, with models estimated for an increasing number of classes until a parsimonious solution with optimal fit across datasets was identified. Model selection was based on the Bayesian Information Criterion and the interpretability of class profiles [47].

### 4.2 Determinants across CSP configurations

Subsequently, descriptive analyses were conducted to examine patterns in determinants for sustainable health and wellbeing system development across the LCA-derived classes. Trust and leadership, which were operationalized as composite measures, were summarized using means and standard deviations. Categorical indicators were summarized using frequencies and percentages within each class. Percentages were calculated based on the number of available responses for each indicator.

## 5 Results

By the end of the collection period, a total of 172 unique CSPs had completed at least one questionnaire, comprising 101 responses in 2023 and 116 in 2024. Based on available data, the annual response rate is estimated at approximately 30–40%. When CSPs responded in both years, the most recent submission was used. The included partnerships covered nearly all municipalities, although the density of CSPs varied across the country. The geographic distribution of the sample shows several concentrations in both urbanized and less populated regions (Appendix C).

### 5.1 Research question 1: Structural and strategic CSP characteristics

Across the full sample, CSPs showed substantial variation in structural and strategic characteristics (Table 2). Structurally, the number of partners and target population size were widely distributed, with clear right-skewed patterns. While most CSPs fall within a relatively limited range (11–31 partners), a small number of markedly larger partnerships extend far beyond this range (maximum: 300). A similar pattern is observed for target population size, where most CSPs fall within a broader central range (8100–325,000), while a small number cover populations of up to several million people (maximum: 3,800,000).

**Table 2:** Overall structural and strategic CSP characteristics (2023–2024).

**Median number of partners involved (IQR)**	20 (11−31)

**Median target population size (IQR)**	65,000 (8100–325,000)
	
**Target population** *Percentage (n) of CSPs focusing on each target group (multiple responses allowed)*	
*Residents (n)*	79.3% (130)
*Clients (n)*	63.6% (105)
*Patients (n)*	60.0% (99)
*Professionals (n)*	81.8% (135)
*Managers/ directors (n)*	58.8% (97)
	
**Prevention orientation** *Percentage (n) of CSPs focusing on each prevention type (multiple responses allowed)*	
*Universal prevention (n)*	43.6% (72)
*Selective prevention (n)*	56.4% (93)
*Indicated prevention (n)*	46.1% (76)
*Care-related prevention (n)*	54.5% (90)
	
**Outcome domains (Quintuple Aim-related)** *Percentage (n) of CSPs focusing on each outcome domain (multiple answers were allowed)*	
*Improving the health of residents (n)*	59.5% (97)
*Improving the quality of care or support (n)*	79.1% (129)
*Reducing the* per capita *costs of care or support (n)*	33.1% (54)
*Improving the well-being of professionals in care or support (n)*	32.5% (26)
*Reducing socio-economic health inequalities (n)*	41.2% (33)
**Involved municipal public health service** *Percentage (n) of CSPs involving at least one municipal public health service*	46.1% (76)

Strategically, many CSPs targeted multiple groups simultaneously, most commonly professionals (81.8%) and residents (79.3%). Engagement in prevention activities was also common, with selective (56.4%) and care-related prevention (54.5%) reported slightly more often than universal prevention (43.6%).

Regarding their intended outcomes, improving the quality of care or support was most frequently reported (79.1%), whereas fewer CSPs explicitly targeted cost reduction (33.1%) or professional wellbeing (32.5%). Together, these patterns illustrate considerable diversity in CSP characteristics.

### 5.2 Research question 2: Two LCA-derived class configurations

A two-class model consistently provided the best result, both in terms of statistical fit, as indicated by the lowest BIC score across imputations for missing values, and conceptual clarity. Classification certainty was high, with average posterior probabilities of at least 0.93 for assigned classes and a normalized entropy value of 0.965, indicating minimal ambiguity in class membership. An overview of the structural and strategic differences between these classes is provided in Table 3.

**Table 3:** LCA-derived class configurations.

	**Class 1: Broadly oriented (*n* = 83) *Share = 0. 474***	**Class 2: Targeted (*n* = 89) *Share = 0.526***
**Number of partners involved** ⁎**C1: *n* = 68; C2: *n* = 67**		
*3–6*	4.00% (3)	15.0% (10)
*7–14*	18.0% (12)	30% (20)
*15–25*	28.0% (19)	37.0% (25)
*26–50*	40.0% (27)	13.0% (9)
*>50*	10.0% (7)	4.00% (3)
**Target population size** ⁎**C1: *n* = 78; C2: *n* = 65**		
*<10,000*	3.00% (2)	55.0% (36)
*10,000-100,000*	31.0% (24)	26.0% (17)
*100,000-500,000*	45.0% (35)	11.0% (7)
*>500,000*	22.0% (17)	8.00% (5)
**Target population: Residents (yes)** ⁎**C1: *n* = 83; C2: *n* = 82**	100% (83)	57.0% (47)
**Prevention orientation: Universal and/or selective** ⁎**C1: n = 82; C2: n = 83**	100% (82)	36.0% (30)
**Outcome domains: Targeting 4–5** **Quintuple Aim elements** ⁎**C1: *n* = 83; C2: *n* = 80**	49% (41)	10% (8)
**Involved municipal public health service** ⁎**C1: n = 83; C2: *n* = 82**	77% (64)	15% (12)

⁎ Percentages were calculated based on the number of responses per variable and may not sum to the total number of CSPs within each class due to missing data.

### 5.3 Class 1 - Broadly oriented CSPs.

CSPs assigned to class 1 are likely to operate at a larger scale than those in class 2, as reflected in both the number of participating organizations and the size of the target population. Nearly half include more than 25 participating organizations (*n* = 34), compared to 17% of class 2 CSPs. Similarly, 67% cover populations of at least 100,000, and 22% exceed 500,000.

Moreover, all class 1 partnerships identify residents as a core target population and engage in universal and/or selective prevention activities. Nearly half address four to five Quintuple Aim domains. In addition, 77% involve municipal public health services (publicly funded regional agencies responsible for health promotion, disease prevention, public health monitoring, and supporting local public health policy and coordination).

### 5.4 Class 2 - Targeted CSPs.

CSPs assigned to class 2 operate at a smaller scale than those in class 1. Most (82%) involve 25 or fewer organizations, and over half (*n* = 36) serve populations under 10,000. Their strategic characteristics also differ: 36% report engagement in universal or selective prevention, and 10% address four or more Quintuple Aim domains. Municipal public health service involvement is reported in 15% of these partnerships.

Together, these classes reflect two distinct organizational patterns within the national CSP landscape:

• Large, multisector, population-oriented networks with broad prevention scope and strong public health involvement.
• Smaller, targeted collaborations focused on specific groups, health conditions, or service pathways.

### 5.5 Research question 3: Determinant patterns across class configurations

Table 4 shows that differences between broadly oriented and targeted CSPs were generally limited for several core determinants, including trust, leadership, shared commitment, and data and learning. Governance structures were also broadly similar across classes, with centralized governance reported in 40% of CSPs and formalization of arrangements regarding tasks and practices being relatively common (63% vs. 61%). In contrast, other forms of formalization were less prevalent across both classes, including financial risk-sharing (35% vs. 21%) and formal arrangements regarding responsibilities for quality (26% vs. 38%). A clearer distinction emerged in coordination across networks, with broadly oriented CSPs more often functioning as a coordinating “network of networks” (29% vs. 15%). Cross-sector co-payment and multi-year contracts were uncommon overall (≤11%), while targeted CSPs more frequently relied on a single payer or involved no payer at all (< two payers: 56% vs. 29%).

**Table 4:** Determinant patterns across class configurations*.*

	**Class 1: Broadly oriented (*n* = 83)** ***Share = 0.4 74***	**Class 2: Targeted (*n* = 89)** ***Share = 0.526***	**Total** **(*n* = 172)**
**Governance and accountability**			
Centralized governance (n) ⁎C1: *n* = 80; C2: *n* = 76	39% (31)	42% (32)	40% (63)
Formalization of arrangements			
*Financial risk-sharing (n)*⁎C1: *n* = 65; C2: *n* = 61	35% (23)	21% (13)	29% (36)
*Only collaborating when benefitting the target population (n)*⁎C1: *n* = 63; C2: *n* = 61	30% (19)	21% (13)	26% (32)
*Practices/ task arrangements within the CSP (n)*⁎C1: *n* = 75; C2: *n* = 71	63% (47)	61% (43)	62% (90)
*Responsibilities for quality across providers (n)*⁎C1: *n* = 66; C2: n = 66	26% (17)	38% (25)	32% (42)
Coordinating “network of networks” (n) ⁎C1: *n* = 83; C2: *n* = 82	29% (24)	15% (12)	22% (36)
**Trust (mean (sd), scale 1–7)**⁎**C1: *n* = 77–75; C2: *n* = 73–71**	5.6 (1.1)	5.9 (0.6)	5.8 (0.9)
**Leadership (mean (sd), scale 1–5)**⁎**C1: *n* = 75–74; C2: *n* = 69**	3.5 (0.6)	3.4 (0.6)	3.5 (0.6)
			
**Data and learning**			
Available CSP-level strategic learning plan (n) ⁎C1: *n* = 77; C2: *n* = 74	60% (46)	58% (43)	59% (89)
Structural data and information exchange (n) ⁎C1: *n* = 74; C2: *n* = 73	66% (49)	67% (49)	67% (98)
Incidental data and information exchange (n) ⁎C1: n = 74; C2: n = 73	27% (20)	25% (18)	26% (38)
			
**Financial arrangements**			
Cross-sector co-payment, aggregated across payer pairs ⁎C1: *n* = 68; C2: *n* = 73			
*Structural co-payment (≥ 1 pair), (n)*	11% (9)	6% (5)	8% (14)
*Incidental co-payment (≥ 1 pair), (n)*	11% (9)	4% (4)	8% (13)
*Co-payment never occurs (n)*	31% (26)	16% (14)	23% (40)
*< Two distinct payers involved (n)*	29% (24)	56% (50)	43% (70)
Alternative funding supporting CSP goals (n) ⁎C1: *n* = 22; C2: *n* = 13	32% (7)	23% (3)	29% (10)
Multi-year contract(s) supporting CSP goals (n) ⁎C1: n = 22; C2: n = 13	9% (2)	15% (2)	11% (4)
			
**Target population engagement**⁎**C1: *n* = 81; C2: *n* = 79**			
Are informed (n)	37% (30)	29% (23)	33% (53)
Are consulted (n)	48% (39)	39% (31)	44% (70)
Provide unsolicited or solicited advice (n)	33% (27)	27% (21)	30% (48)
Involved in shaping activities (n)	47% (38)	34% (27)	41% (65)
Involved in implementing activities (n)	36% (29)	38% (30)	37% (59)
Involved in governance decision-making (n)	21% (17)	14% (11)	18% (28)
Not involved in the CSP (n)	2% (2)	11% (9)	7% (11)
			
**Alignment with policy**			
Alignment with GALA policy: Integrated Care approaches ⁎C1: *n* = 59; C2: *n* = 47			
*No alignment (0 approaches), (n)*	22% (13)	70% (33)	43% (46)
*Partial alignment (1–4 approaches), (n)*	44% (26)	30% (14)	37% (40)
*Full alignment (all approaches), (n)*	34% (20)	0% (0)	19% (20)
Alignment with GALA policy: Thematic priorities ⁎C1: n = 59; C2: n = 47			
*No alignment (0 priorities), (n)*	3% (2)	49% (23)	24% (25)
*Partial alignment (1–5 priorities), (n)*	65% (38)	47% (22)	57% (60)
*Full alignment (all priorities), (n)*	32% (19)	4% (2)	20% (21)
Alignment with IZA policy: Target population ⁎C1: *n* = 81; C2: *n* = 68			
*Older adults with health vulnerabilities (n)*	90% (73)	76% (52)	84% (125)
*People with (risk of) cancer (n)*	40% (32)	31% (21)	36% (53)
*People with (risk of) cardiovascular disease (n)*	51% (41)	38% (26)	45% (67)
*People with limited health literacy (n)*	84% (68)	57% (39)	72% (107)
*People with mental health issues (n)*	73% (59)	50% (34)	62% (93)
			
**Shared commitment**⁎**C1: *n* = 76; C2: *n* = 69**			
Extremely good (n)	4% (3)	1% (1)	3% (4)
Very good (n)	57% (43)	51% (35)	54% (78)
Somewhat good (n)	36% (27)	45% (31)	40% (58)
Not so good (n)	4% (3)	3% (2)	3% (5)
			
**Resource sufficiency, non-financial (% everything or most of what the CSP needs)**			
Convening power (n) ⁎C1: *n* = 72; C2: *n* = 68	93% (67)	82% (56)	87% (123)
Data and information (n) ⁎C1: *n* = 72; C2: *n* = 67	39% (28)	43% (29)	41% (57)
Skills and expertise (n) ⁎C1: n = 72; C2: *n* = 68	87% (63)	84% (57)	86% (120)
Target population relations (n) ⁎C1: *n* = 71; C2: *n* = 68	53% (38)	75% (51)	65% (89)

⁎ Percentages were calculated based on the number of responses per variable and may not sum to the total number of CSPs within each class due to missing data.

Further variation was observed in target population engagement and relations, and policy alignment. Broadly oriented CSPs more often involved target populations in shaping activities (47% vs. 34%), whereas targeted CSPs more frequently reported no involvement (11% vs. 2%). At the same time, perceived sufficiency of target population relations was higher among targeted CSPs than among broadly oriented CSPs (75% vs. 53%). In addition, broadly oriented CSPs showed substantially higher alignment with GALA integrated care approaches (no alignment: 22% vs. 70%) and thematic priorities (full alignment: 32% vs. 4%), as well as more consistent alignment with IZA target populations (e.g., limited health literacy: 84% vs. 57%).

Finally, while perceived sufficiency of skills and expertise (86%) and convening power (87%) were relatively high across both classes, perceived sufficiency of data and information that would support their efforts was notably lower (41%).

## 6 Discussion

This study provides the first empirical, national-level overview of CSPs in the Netherlands and shows that regional collaboration is taking shape through distinct yet coexisting configurations within a shared policy context. The identification of two class configurations, broadly oriented and targeted CSPs, highlights that national policy ambitions are not translated into a single dominant collaboration model, but rather into a layered landscape of partnerships with different roles, scopes, and coordination demands. The variation in CSP configurations observed in this study is consistent with findings from a systematic review by Alderwick et al. [6], which documents substantial heterogeneity in the scope and focus of cross-sector collaborations, ranging from broad population health partnerships to more narrowly defined, service- or population-specific initiatives.

Notably, both types are observed here within the Dutch national policy context. This pattern most likely reflects the collaboration landscape shaped by the layering of IZA-level regional structures onto pre-existing, often smaller, subregional networks. While not unexpected, this raises important questions about how roles and responsibilities are distributed across levels of collaboration. Without clear alignment and role differentiation, this layered structure risks increasing coordination complexity [48]. Internationally, reforms such as England's Integrated Care Systems have similarly steered regional collaboration toward broader, population-based configurations, while pre-existing networks continue to operate within these broader system structures 12., 32.. This was accompanied by substantial organizational restructuring, which disrupted existing relationships, created uncertainty about roles and responsibilities, and diverted capacity away from ongoing work, suggesting that layering collaborative structures does not automatically lead to better integration and may require stronger alignment across levels of governance [21].

The characteristics of the class configurations in this study suggest a potential division of functions, in which broader regional CSPs articulate system-level ambitions and population-oriented strategies, while more targeted partnerships focus on specific populations, conditions, or service pathways. At the same time, both configurations show considerable similarity across determinants for sustainable health and wellbeing system development. Differences were most apparent in alignment with current policy agendas. This pattern is further supported by the timing of their initiation, as broadly oriented CSPs more predominantly emerged during more recent policy periods, particularly following the introduction of IZA and GALA. Beyond this, contrasts primarily reflect variation in strategic orientation and the associated coordination demands. Broadly oriented CSPs more often function as structures linking multiple organizations and existing partnerships, and more frequently involve payers from multiple sectors. Network governance theory highlights that coordination becomes more demanding as networks encompass a larger and more diverse set of participants and relationships, increasing the complexity of coordinating activities and interests at the level of the network as a whole [9]. These differences in scope and associated coordination demands are likely to be accompanied by different needs for network-level competencies. However, the determinant profiles observed in this study were largely similar. This suggests that, despite their differences, both configurations may face common challenges related to the conditions needed to support their development, although these may be more consequential for broadly oriented CSPs, given their larger scale, broader scope, and associated coordination demands.

Across both configurations, trust, leadership, and shared commitment were relatively well established, whereas accountability formalization, financial arrangements, and data availability remained less developed. From a developmental perspective, this pattern is notable because the areas in which greater challenges remain largely concern conditions needed to embed collaboration more structurally and support its institutionalization beyond a primarily relational foundation 31., 49.. The findings therefore suggest that, despite their different configurations, CSPs may face similar challenges in translating established relational foundations into more structurally embedded coordination. This is consistent with earlier large-scale analyses in the United States, where multisector partnerships were often found to lack the characteristics required to transform regional health systems, particularly regarding governance arrangements, outcome measurement, and sustainable financing, as well as with findings from Ontario Health Teams and Belgian integrated care initiatives 13., 34., 35., 49., 50.. The persistence of these challenges across contexts and partnership configurations suggests that they may not be explained by partnership characteristics alone. Instead, they may reflect a broader misalignment between emerging cross-sector collaborative practices and the institutional environments in which they are embedded, as the wider system context can create both opportunities and constraints for the development and functioning of collaboration [36]. In particular, limited progress in accountability formalization, sustainable financing, and data infrastructure points to areas where individual partnerships have limited capacity to effect change, as these are also shaped by system-level structures and regulations. Without changes in payment or regulatory systems, CSPs are likely to struggle to move beyond optimizing existing practices toward more transformative forms of system change [49]. Overcoming this gap may therefore require improved coordination across governance levels and more active involvement of system-level actors to help establish and formalize conditions for sustainable cross-sector collaboration, aligning bottom-up integrative practices with top-down structures and incentives that enable coordination and integration 36., 51..

### 6.1 Methodological considerations

Several considerations are important when interpreting the findings. First, while the CSP inclusion criteria provided a clear and pragmatic basis for analysis, partnership boundaries and populations are inherently dynamic, and no single definitive definition has been established. In addition, because no national registry of Dutch CSPs exists, the representativeness of the sample is uncertain and certain types of partnerships may be under- or overrepresented, particularly those more visible, networked, or engaged in national policy programs. The absence of such a registry is closely related to the decentralized way in which CSPs have developed in the Dutch context, shaped by regional contexts, existing relations, and local negotiation rather than centrally mandated structures. As a result, the sample necessarily built on previously identified collaborations supplemented through additional outreach efforts. While this limits representativeness in a statistical sense, it also reflects the evolving and locally embedded nature of Dutch CSPs.

Second, model fit statistics and high classification certainty indicate that the two-class solution provides a strong and parsimonious representation of heterogeneity in the data. In addition, despite introduced uncertainty through estimation, the consistency of results across imputed datasets suggests that the overall class structure is stable. However, as with all LCA-based typologies, the resulting classes should be interpreted as configurations that capture dominant patterns rather than sharp boundaries or fixed developmental pathways. Furthermore, although the LCA identified distinct configurations, both represent CSPs coordinating multiple autonomous organizations around complex health and wellbeing challenges. The analysis therefore distinguishes configurations within an already demanding form of collaboration and, as a result, does not capture the full range of coordination demands that exists across interorganizational arrangements.

Third, the survey was originally developed for broader monitoring and evaluation purposes rather than specifically for this study; thus, the selected indicators reflect a pragmatic use of available data. Moreover, no standardized quantitative operationalization of the guiding principles exists, therefore the results should be interpreted as an indicative assessment that necessarily simplifies complex and context-dependent collaborative processes. In addition, the reliance on self-reported data introduces potential perceptual and reporting biases, particularly for relational constructs such as trust and leadership. Because data were generally provided by a single representative on behalf of each CSP, findings reflect individual perceptions that may differ across stakeholders within the same collaboration.

Finally, the cross-sectional design offers a snapshot of CSPs at a specific moment in time and does not capture developmental dynamics or causal relationships. Caution is therefore warranted when interpreting the findings over time or generalizing them to other policy settings, especially where institutional roles and system structures differ from the Dutch context.

### 6.2 Implications and future research

The findings, which largely align with existing international evidence, offer policymakers and regional stakeholders a clearer picture of the national CSP landscape, enabling more targeted forms of support, benchmarking, and peer learning across partnerships. For policymakers, the findings highlight that CSPs operate in distinct configurations, calling for support that accounts for differences in scope, scale, and coordination demands. At the same time, shared challenges in financing, accountability formalization, and data availability highlight areas where support at the level of individual partnerships may need to be complemented by support at the system level. For practice, the results underscore that although strong relational foundations are often in place, sustained cross-sector collaboration also requires greater structural embedding. This calls for further development within partnerships themselves, alongside the involvement of policymakers and other system-level actors in addressing financing, regulatory, and other structural barriers to sustained cross-sector collaboration. However, strengthening cross-sector collaboration should not be regarded as an end in itself. Ultimately, its value lies in whether it enables organizations to better address population needs and contributes to more coordinated and responsive services and support.

Building on these findings, several important directions for future research emerge. First, longitudinal studies are needed to examine how CSPs develop over time, including whether and how their configurations change and how relational and structural conditions for collaboration develop alongside them. Second, linking CSP configurations to measures of collaboration effectiveness and relevant outcomes would provide insight into whether specific structural or strategic characteristics are associated with greater effectiveness or stronger impacts, as well as whether different configurations can be similarly effective in achieving their respective goals while differing in the network-level competencies needed to address their coordination demands. Finally, more large-scale international comparative research could assess whether the patterns observed in the Netherlands are also found in other countries pursuing regional collaborative approaches in the organization of health and wellbeing services, or whether they reflect specific features of the Dutch context. Such research would enable cross-country learning about what works, for whom, and under what conditions in addressing complex health system challenges.

## 7 Conclusion

This study provides a national-level overview of cross-sector partnerships for health and wellbeing in the Netherlands, showing that regional collaboration is taking shape through multiple, coexisting configurations within a shared policy context. While partnerships differ substantially in strategic orientation and coordination demands, the similarities observed across configurations suggest that many CSPs are navigating similar challenges in moving beyond relational foundations toward more structurally embedded collaboration, particularly in accountability formalization, sustainable financing, and the development of data infrastructure. By empirically distinguishing these patterns, the study advances understanding of how regional collaboration currently unfolds in practice and provides a foundation for future longitudinal and comparative research. The extent to which CSPs can contribute to the transformation toward sustainable health and wellbeing systems will depend on how these partnerships develop and consolidate within their wider system contexts.

## CRediT authorship contribution statement

**Sam Hundersmarck:** Writing – review & editing, Writing – original draft, Visualization, Validation, Resources, Project administration, Methodology, Investigation, Formal analysis, Data curation, Conceptualization. **Miel Vugts:** Writing – review & editing, Visualization, Validation, Software, Methodology, Investigation, Formal analysis, Data curation, Conceptualization. **Annefrans van Ede:** Writing – review & editing, Supervision, Methodology, Conceptualization. **Marc Bruijnzeels:** Writing – review & editing, Supervision, Methodology, Conceptualization. **Natascha van Vooren:** Writing – review & editing, Supervision, Resources, Methodology, Investigation, Conceptualization.

## **Funding**

This research did not receive any specific grant from funding agencies in the public, commercial, or not-for-profit sectors.

## **Declaration of competing interest**

The authors declare that they have no known competing financial interests or personal relationships that could have appeared to influence the work reported in this paper.

## Appendix A Supplementary data

Supplementary File 1. Supplementary materials containing Appendices A-D, including additional methodological details and supporting data.
