# Supplementary material for: Transforming health systems: Mapping the landscape with 172 regional cross-sector partnerships in the Netherlands
Source: Health Policy Open. 2026 Oct 23;11:100192. doi: 10.1016/j.hpopen.2026.100192 (PMC13769114; doi:10.1016/j.hpopen.2026.100192)
Supplement: Supplementary file 1 [file mmc1.docx]

Appendix A. RIVM Survey on Cross-sector Partnerships (2023-2024)

**Identification of the partnership**

1. What is the name of the cross-sector partnership (CSP)?
2. What is your primary role or position within this CSP?

- Network manager / program manager
- Other, namely: _______

3. Is this CSP currently active?

- Yes, this initiative is currently active
- No, this initiative has merged into another initiative
- No, this initiative has ended

4. *(If merged)* What is the name of the initiative into which it has merged?
5. What was the start date of the CSP in its current form?
   (If the exact day or month is unknown, please enter “01” for the missing value.)
6. Has the CSP had one or more predecessor initiatives?

- Yes
- No

7. *(If yes)* Under what name was the first predecessor known?
8. *(If yes)* In which year did the first predecessor start?
9. Is there a website where information about the current CSP can be found?
   Please provide the URL.

**Eligibility criteria**

According to the study definition, a CSP meets the following criteria:

- Three or more organisations participate
- Organisations from at least two of the following domains participate:
  - social domain
  - long-term care
  - other healthcare
- There is a shared ambition to improve care or support for residents in a defined geographic area
- There is a shared intention to collaborate for more than one year
- The collaboration includes at least two concrete joint activities (e.g. projects or interventions) focused on the needs and functioning of residents or patients

10. Do all of the above criteria apply to the CSP you represent?

- Yes
- No

11. *(If no)* Which criteria are not met?

**Relation to National Policy Frameworks**

12. Does the CSP fall within an IZA regional plan?

- Yes
- No

13. *(If yes)* Within which IZA region(s) does the CSP fall?
14. How does the CSP relate to the IZA regional plan in this region?

- The CSP implements the entire regional plan
- The CSP contributes to part of the regional plan
- The CSP operates alongside the regional plan
- Other, namely: _______

15. To which themes or care pathways from the Healthy and Active Living Agreement (GALA) do the activities of the CSP relate?
    (Multiple answers possible)

- Theme: Reducing health inequalities
- Theme: A healthy physical environment that encourages physical activity and social interaction
- Theme: Strengthening the social base
- Theme: A healthy lifestyle
- Theme: Strengthening mental resilience and mental health
- Theme: Healthy ageing
- Care pathway: Healthy Start (Kansrijke Start)
- Care pathway: Social prescribing / Wellbeing on prescription
- Care pathway: Combined Lifestyle Intervention (GLI)
- Care pathway: Tackling childhood overweight and obesity / Healthy Weight for Children (KnGG)
- Care pathway: Fall prevention
- None of the above

**Position relative to other partnerships**

16. Which of the following best describes the role of the CSP?

- The CSP primarily coordinates collaboration between organizations
- The CSP primarily coordinates collaboration between multiple networks of organizations

17. *(If applicable)* Which networks are connected or coordinated by the CSP?
    [Open text]

**Ambition and objectives**

18. What is the shared overarching ambition (reason for existence) of the CSP?
    [Open text]
19. In which of the following areas has the CSP formulated concrete goals?
    (Multiple answers possible)

- Improving the health of residents
- Improving the quality of care and/or support
- Containing the costs of care and/or support per resident
- Improving the well-being of professionals in care and/or support
- Reducing socioeconomic health inequalities
- Mitigating workforce shortages
- Improving accessibility of care and/or support
- Other, namely: _______

**Prevention orientation**

20. Which forms of prevention does the CSP address?
    (Multiple answers possible)

- Universal prevention (aimed at the entire population, including people without risk or condition)
- Selective prevention (aimed at risk groups)
- Indicated prevention (aimed at individuals with elevated risk)
- Care-related prevention (aimed at preventing deterioration among people already receiving care)

**Broad health concepts**

21. Does the CSP work from a broad concept of health?
    (Multiple answers possible)

- Positive Health
- Strengthened Primary Care
- A shift from disease and care towards health and behaviour
- Vitality and lifestyle
- Recovery-oriented approach
- Reablement
- Other, namely: _______
- No, none of these

**Target population**

22. Which population(s) does the CSP focus on?
    [Open text]
23. Which type(s) of population does the CSP focus on?
    (Multiple answers possible)

- Residents
- Clients
- Patients
- Professionals
- Administrators or managers
- Other, namely: _______

24. Which priority groups from the Integrated Care Agreement (IZA) partially or fully overlap with the population targeted by the CSP?
    (Multiple answers possible)

- People with limited health literacy
- People with mental health problems
- Frail older adults
- People at increased risk of cancer
- People at increased risk of cardiovascular disease
- Other, namely: _______

25. What is the estimated size of the total population targeted by the CSP?
    [Numeric entry]
26. Within which geographic area is the targeted population located?
    (Select applicable region(s) or describe)
27. If applicable, in which neighborhoods or districts is the population located?
    [Open text]

**Participating organizations**

28. Approximately how many organizations participate in the CSP?
    (An estimate is acceptable.)
    [Numeric entry]
29. For each type of organization listed below, please indicate:

a) whether the organization contributes to achieving the ambition or activities of the CSP
b) whether the organization is represented in the steering committee or board of the CSP

(Response options per column: Yes / No)

**Healthcare domain**

- Health insurers
- Care offices
- General practice
- Hospital (academic)
- Hospital (general/peripheral)
- Mental healthcare (including addiction care)
- Long-term care organizations (including nursing and residential care)
- District nursing / home care
- Paramedical care (e.g. physiotherapy, dietetics, occupational therapy)
- Pharmacy / pharmaceutical care
- Maternity care
- Independent clinics
- Patient representation
- Other healthcare organizations, namely: _______

**Social domain**

- Municipalities
- Public health service (GGD)
- Welfare organizations / social work
- Youth healthcare
- Lifestyle coaches
- Debt support services
- Daily living support / participation support
- Client representation
- Other social domain organizations, namely: _______

**Knowledge and education**

- Primary and/or secondary education
- Vocational education
- Universities or academic institutions
- Other, namely: _______

**Other sectors**

- Employers
- Housing corporations
- Sports organizations
- Volunteer organizations
- Resident associations
- Umbrella organizations / advocacy groups
- Other, namely: _______

**Resident involvement**

30. In what way are residents, patients, or clients involved in the CSP?
    (Multiple answers possible)

- Not involved
- Informed about activities
- Consulted for input
- Provide advice
- Involved in co-creation of activities
- Involved in implementation
- Involved in decision-making

**Payers and financial collaboration**

31. Which municipalities are involved in the CSP?
    [Open text or selection]
32. Which health insurers and/or care offices are involved in the CSP?
    [Open text or selection]
33. Do these organizations exchange financial resources related to the CSP (e.g., co-payment or shared savings)?

For each combination indicate the frequency:
(Never / Occasionally / Structurally)

- Municipality ↔ Health insurer
- Health insurer ↔ Care office
- Care office ↔ Municipality

34. Please briefly describe how financial resources are exchanged between payers within the CSP.
    [Open text]

**Governance**

35. Which governance model best describes the CSP?

- Shared governance among partners
- One lead organization coordinates the partnership
- A separate network administrative organization (NAO) coordinates the partnership
- Other, namely: _______

36. To what extent have formal agreements been made about the following topics?
    (Response options: Absent / Informal / Formal)

- Roles and tasks of participating organizations
- Agreements about collaboration boundaries (e.g. competition considerations)
- Responsibilities for quality of care/support across organizations
- Financial risk-sharing in case of service changes

37. If formal agreements have been made regarding financial risk-sharing, what types of payment arrangements have been established within the CSP?

(Multiple answers possible)

- Adjustment of payment arrangements based on a Dutch Healthcare Authority (NZa) scheme
- Multi-year contract(s)
- Alternative payment models (e.g. outcome-based payments, shared savings, bundled payments/integrated financing, or population-based payment)
- I don’t know
- Other, namely: _______

**Interorganizational Interaction and Learning**

38. For each of the following forms of collaboration, indicate how often this occurs between two or more organizations within the CSP.

Response options:

- Never
- Occasionally
- Structurally (regularly)

a. Providing care and support jointly
b. Exchanging data, information, or knowledge
c. Exchanging financial resources

39. If knowledge or information is exchanged: does this include exchange for the purpose of learning or knowledge development between managers, administrators, or professionals?

- Yes
- No
- Don’t know

40. Has this learning led to adjustments in working methods within the CSP?

- No
- Yes, at the operational level
- Yes, at the tactical (management) level
- Yes, at the strategic (board) level

41. Is there a plan or strategy to facilitate learning among participants in the CSP?

- Yes
- No
- Don’t know

**Trust between partners**

Please indicate the extent to which you agree with the following statements.

Response scale:
1 = Strongly disagree
2 = Disagree
3 = Somewhat disagree
4 = Neither agree nor disagree
5 = Somewhat agree
6 = Agree
7 = Strongly agree

42. I expect other parties within the CSP to tell the truth.
43. I am confident that I can fully trust other parties within the CSP.
44. I expect other parties within the CSP to keep their commitments.

**Collaborative capacity**

Considering the organizations and individuals in the CSP:
By working together, how well are participants able to:

Response scale:
1 = Not at all well
2 = Not very well
3 = Moderately well
4 = Very well
5 = Extremely well

45. Find different ways to solve problems
46. Take into account the perspectives and priorities of those affected
47. Set goals that are widely understood and supported
48. Respond to the needs of the target population
49. Implement strategies likely to work best
50. Mobilize individuals and organizations in the community
51. Carry out integrated activities connecting services or systems

**Leadership effectiveness**

Please rate the overall effectiveness of leadership within the CSP.

Response scale:
1 = Not at all effective
2 = Not very effective
3 = Moderately effective
4 = Very effective
5 = Extremely effective
6 = Don’t know

52. Taking responsibility for the CSP
53. Motivating participants involved in the collaboration
54. Enabling participants to contribute
55. Recruiting relevant organizations and stakeholders

**Availability of resources**

To what extent does the CSP have sufficient access to the following resources to function effectively?

Response scale:
1 = None (0–5%)
2 = Very little (5–20%)
3 = Some (20–50%)
4 = Most (50–95%)
5 = All (95–100%)
6 = Don’t know

56. Skills and expertise
57. Data and information
58. Relationships with representatives of the target population
59. Influence and ability to bring people together for meetings and activities

**Use of partners’ resources**

How well does the CSP make use of the following resources contributed by participating organizations?

Response scale:
1 = Not at all well
2 = Not very well
3 = Moderately well
4 = Very well
5 = Extremely well

60. Financial resources contributed by participants
61. In-kind contributions (e.g., expertise, information, facilities, materials)
62. Time contributed by participants

**Perceived effectiveness**

How effective has the CSP been so far in achieving its goals in the following domains?

Response scale:
1 = Not at all effective
2 = Slightly effective
3 = Moderately effective
4 = Very effective
5 = Extremely effective
6 = Don’t know

63. Improving the health of residents
64. Improving the quality of care and/or support
65. Containing the costs of care and/or support per resident
66. Improving the well-being of professionals in care and/or support
67. Reducing socioeconomic health inequalities
68. Mitigating workforce shortages
69. Improving accessibility of care and/or support

**Organizational change within participating organizations**

Since the start of the CSP, to what extent have the following changed within participating organizations in line with the partnership’s goals?

Response scale:
1 = None or very little
2 = Little
3 = Moderate
4 = Much
5 = Very much

70. Strategic plans of organizations
71. Implementation of strategic plans
72. Services offered by organizations
73. Staffing and workforce allocation
74. Governance structures
75. Organizational vision or mission

**Optional respondent information**

76. Name
77. Email address
78. Organization
79. Position within the CSP
80. May we contact you for follow-up research? (Yes/No)

**Final questions**

81. Do you have any additional comments about the CSP?
    [Open text]
82. Do you have feedback about this questionnaire?
    [Open text]

Appendix B. Determinant measurement and operationalization

*Table B1. Determinants included in descriptive analysis*

| Determinant | Survey operationalization | Operational indicators  (survey items) | Validity assessment |
| --- | --- | --- | --- |
| Governance and accountability | Formalization of agreements and governance mode | Formal agreements on tasks and responsibilities, quality, alignment of financial incentives (36); network governance mode (35); network of networks (16) | N.A.* |
| Trust | Mutual expectations of honesty and reliability | Responses to items on honesty (42), keeping promises (44), confidence in other parties (43) | Simons & Peterson^45^; Cronbach’s α = 0.88 (n = 146) |
| Leadership | Effectiveness of leadership across sectors | Ratings of leadership on responsibility (52), inspiration (53), enabling contributions (54), recruiting partners (55) | Short-PSAT (Cramm et al.^36^);  Cronbach’s α = 0.81 (n = 143) |
| Data and learning | Knowledge exchange and adaptation | Presence of data exchange (38b/39), existence of a learning plan (41) | N.A.* |
| Financial arrangements | Supporting cross-sector financial arrangements | Existence of financial exchange between payers (31-33) and supporting payment arrangements (37) | N.A.* |
| Target population engagement | Engagement of target population | Types of engagement, i.e. representation, advising, and co-creation roles in activities (30) | N.A.* |
| Alignment with policy | Connection to national agreements | Reported links to IZA target populations (24) and GALA policies (15) | N.A.* |
| Shared commitment | Shared understanding and support for goals | Perceived shared understanding and support for CSP goals (47) | Single item from the partnership synergy scale (Cramm et al.^36^) |
| Resource sufficiency (non-financial) | Sufficiency of non-financial resources | Perceived sufficiency of skills (56), information (57), relationships (58), convening power (59) | Separate items from short-PSAT, low internal consistency (Cronbach’s α = 0.52; n = 139) |

*N.A.: Validated Likert-type scales were not applied, since the items assessed objectively observable phenomena rather than latent constructs

Appendix C. Geographic distribution of sampled CSPs

*Figure C1. Geographic distribution of sampled CSPs across Dutch municipalities (2023–2024)*

Appendix D. Determinant item plots across LCA-derived classes

Plots of the chosen determinants across LCA-derived classes and CSPs in total:

*Governance*

*Trust*

*Leadership*

*Data and learning*

*Financial arrangements*

*Target population engagement*

*Alignment with policy*

*Shared commitment*

*Resource sufficiency (non-financial)*
